# Physicochemical properties associated with the presence of *Burkholderia pseudomallei* in small ruminant farm water supplies in Peninsular Malaysia

**DOI:** 10.1007/s10661-018-6613-7

**Published:** 2018-03-22

**Authors:** Hassan Ismail Musa, Latiffah Hassan, Zulkifli Hj. Shamsuddin, Chandrawathani Panchadcharam, Zunita Zakaria, Saleha Abdul Aziz

**Affiliations:** 1 Faculty of Veterinary Medicine, Universiti Putra Malaysia, Serdang, Selangor Malaysia; 2 Department of Veterinary Public Health and Preventive Medicine, Faculty of Veterinary Medicine, University of Maiduguri, Maiduguri, Borno State Nigeria; 3 Department of Land Management, Faculty of Agriculture, Universiti Putra Malaysia, Serdang, Selangor Malaysia; 4 Veterinary Research Institute, 59 Jalan Sultan Azlan Shah, Ipoh, Perak Malaysia

**Keywords:** *Burkholderia pseudomallei*, Physicochemical, Water, Environment, Small ruminants, Malaysia

## Abstract

*Burkholderia pseudomallei* causes melioidosis, a life-threatening infection in both humans and animals. Water is an important reservoir of the bacteria and may serve as a source of environmental contamination leading to infection. *B. pseudomallei* has an unusual ability to survive in water for a long period. This paper investigates physicochemical properties of water associated with the presence of *B*. *pseudomallei* in water supply in small ruminant farms in Peninsular Malaysia. Physicochemical properties of water samples taken from small ruminant farms that included temperature, pH, dissolved oxygen (DO_2_), optical density (OD), and chemical oxygen demand (COD) were measured after which the samples were cultured for *B. pseudomallei.* Multivariable logistic regression model revealed that slightly acidic water pH and higher COD level were significantly associated with the likelihood of the *B. pseudomallei* presence in the water.

## Introduction

*Burkholderia pseudomallei* is the causal agent for melioidosis, a life-threatening infection of both humans and animals (How and Liam 2006; Limmathurotsakul et al. 2012). Melioidosis is an emerging disease among livestock in Malaysia, a country considered endemic for the disease (Hassan et al. 2010). Water is one of the important environmental reservoirs of *B. pseudomallei* and may serve as a source of contamination (Inglis et al. 2000) and infection for humans and animals since the agent possesses ability to survive even in distilled water for up to 16 years (Pumpuang et al. 2011). In Taiwan, *B. pseudomallei*-contaminated water was implicated as a source of the disease among humans (Dai et al. 2012). Similarly, contaminated water supplies have also been implicated in outbreaks of melioidosis in humans in other endemic areas (Inglis et al. 2000; Currie et al. 2001; Limmathurotsakul et al. 2014). Survival of *B. pseudomallei* in liquid suspension has been shown to be influenced by factors such as temperature (Robertson et al. 2010), type of suspension medium (Shams et al. 2007), concentration of salt in the medium (Hicks et al. 2000; Pumirat et al. 2014), and pH of the medium (Inglis et al. 2003; Inglis and Sagripanti 2006). In addition, physicochemical parameters of water that included temperature, low pH, salinity, iron contents, phosphate, and turbidity have also been reported to be associated with the presence of *B. pseudomallei* in water (Draper et al. 2010).

Parameters that might influence the presence and persistence of the agent in water in livestock farms in Malaysia have not been studied. In this study, we investigated the presence of the agent in small ruminant farm water supplies and the physicochemical properties of the water associated with the presence of *B. pseudomallei*. Understanding relationships between the physicochemical properties and the presence of *B*. *pseudomallei* in water from the farm environment may improve understanding of the environmental epidemiology of *B. pseudomallei* and may enhance disease control strategies among livestock in these areas.

## Materials and methods

### Study design

**Fig. 1:**
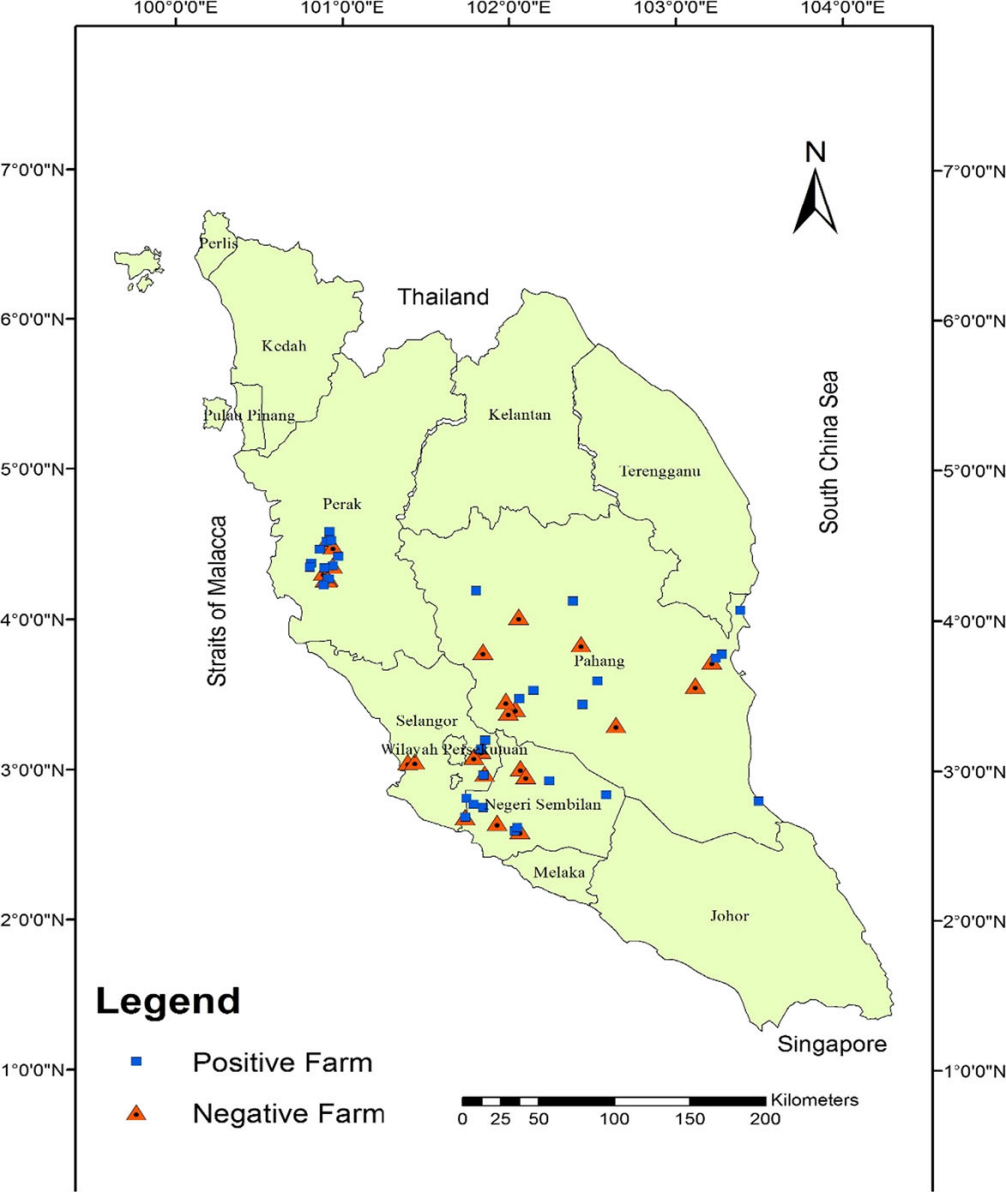
Map of Peninsular Malaysia showing locations of small ruminant farms where water samples were obtained for the study

The study design for this project has been described in detail elsewhere (Musa et al. 2016). Briefly, goat and sheep farms were selected from four states in Peninsular Malaysia which included Negeri Sembilan, Pahang, Perak, and Selangor. Letters requesting for the farmers’ participation in the study were sent to the individual farmers before commencement of the study. Only those who indicated their willingness and agreed to participate were visited. The study farms were selected from animal disease surveillance database obtained from the Department of Veterinary Services (DVS), Putrajaya and Veterinary Research Institute (VRI), Ipoh. The database also serves as official record for national animal disease surveillance program of which melioidosis was considered as a multiple species disease. A map of Peninsular Malaysia showing the locations and status of small ruminant farms from which water samples were collected is shown in Fig. 1.

### Sample collection

The study farms were classified as intensive, semi-intensive, and extensively managed farms for the purpose of environmental water sample collection. Farms where animals were localized in housing units were categorized as intensively managed and water samples were collected from the drinking water sources such as water taps, boreholes, and wells located in the farms’ premise. For farms categorized as semi-intensive and extensive, water samples were taken from drinking water sources in the farms’ premise as well as from any other environmental water source in the grazing areas. Water sources that included ponds, pools, rivers, and dams to which animals in the farm have access to were sampled when available.

A modified format of the sampling procedure described by Inglis et al. (2004) was adopted for the water sample collection. For collection of tap-borne water samples, the pipe outlets were disinfected using 70% alcohol and the water allowed to run for about 5 min then the samples collected into sterile containers. For collection of water samples from environmental surface water sources such as ponds and rivers, sterile sample bottles were submerged to depths of about 10 cm below the surface then opened, filled, and closed before removal. The samples were kept in Iceman’s cool box away from direct sunlight and transported to the Bacteriology Laboratory, Faculty of Veterinary Medicine Universiti Putra Malaysia, for processing.

### Measurements of physicochemical properties of samples

Temperatures and dissolved oxygen (DO_2_) contents of the water samples were measured simultaneously at the point of collection using dissolved oxygen pen (850045 model, Sper Scientific Ltd, Scottsdale, USA) by inserting the pen’s probe to a depth of about 20 cm below the water surface and recording the readings from the display. The pH of samples was measured using a portable pH meter (Hanna Instruments, Australia). The sample chemical oxygen demand was measured using the COD meter (Hanna Instrument, Australia). The optical density (ODs) of sample was measured using a spectrophotometer.

### Culture and identification of *B. pseudomallei*

The water samples were cultured according to a modified formats of the procedures described by Mayo et al. (2011) and Zanetti et al. (2000). One hundred milliliters of water samples was filtered through a sterile 0.42-μm pore membrane filter mounted on a vacuum pump. After filtration, the membrane filters were first immersed in 15 ml of Ashdown’s selective broth then placed on Ashdown’s agar. Both broths and the plates were incubated at 37 °C. Broths were examined every 24 h for 7 days for pellicle formation. The pellicles when present were washed and sub-cultured on Ashdown’s agar. When pellicle is not visible, 5 μl of broth was taken from the topmost layer on the surface, plated on Ashdown agar, and incubated as above. The agar and broth were examined daily for 4–7 days for growth of the characteristic *B*. *pseudomallei* colonies. Suspect colonies were sub-cultured on blood agar and screened using catalase and oxidase tests. The presumptive isolates were further screened using API 20NE kits according to the manufacturer’s instructions. Final confirmation of the isolates (API 20NE-positive isolates) was done using PCR amplification as described by Brook et al. (1997). The amplifications were performed using primers (PPM3 and PPM4) selected from the 16S rRNA region of *B. pseudomallei.* The sequences were from position 452 to 472 (forward primer) 5′ AATCATTCTGGCT AATACCCG 3′ and position 1023 to 1042 (reverse primer) 5′ CGGTTCTCTTTCGAGCTCG 3′. The DNA templates were prepared from the presumptive isolates by taking 1 ml of the aliquots of suspected broth cultures and centrifuged at 12000 rpm for 2 min so that the cells are pelleted. Then 0.1 ml of sterile distilled water was added to the pellets and the suspension heated at 94 °C for 10 min in boiling water bath. The suspensions were centrifuged again at 8000 rpm for 2 min and supernatant fluid was collected and kept at − 20 °C until use. Five 1 μl of the supernatant fluid were used per each 50 μl PCR reaction.

The PCR reaction mix after optimization consisted of dNTPs (4 × 2.5 mmol/l), 1× PCR buffer containing magnesium chloride (15 mmol/l), sterile glycerol (14%), and 0.5–1.0 μmol/l of each primer. The reaction mix was made up to 50 μl with sterile RNAase-free distilled water. One microliter of Taq DNA polymerase (Biobasic) diluted 1:5 with 10× buffer was added to each tube, mixed well, and overlaid with 60 μ1 of sterile paraffin oil. The amplification process was carried out using a Thermal Cycler (MyCyler®, Bio Rad, US). The PCR protocol consisted of 30 cycles of 1 min at 94 °C, 30 s at 54 °C, and 2 min at 72 °C, with a final extension step of 10 min at 72 °C. Products were visualized by electrophoresis on a 1.0% agarose gel (Bio Rad) stained with ethidium bromide (0.1%). Three replicates of isolates were used in each reaction.

### Data analysis

The data obtained from the study were managed in Microsoft Excel. Chi-squared test was used to determine association between the presence of *B. pseudomallei* in water and categorical variables of the farms such as management type, state where farm was located, type of small ruminant in the farm, and source of the sample. The sources of samples were re-categorized as underground water (boreholes, well) and surface water (pond, river, pool, dam, tap water) to enable analysis. For continuous variables, the water parameters were compared between positive and negative samples using independent *t* test. Correlational analysis was used to assess collinearity between the continuous independent variables and the variable OD was found to have significant correlations with almost all other independent variables. Therefore, this variable (OD) was excluded from our model construction. A multivariable logistic regression model was constructed using a backward stepwise method in which the water parameters with a univariable level of significance *p* < 0.25 were selected for inclusion in the base model, and variables were excluded if the *p* value was > 0.05 and did not meaningfully alter the point estimates of the remaining variables. The overall goodness-of-fit of the model to the data was examined using the Hosmer-Lemeshow test. The statistical analyses were performed using SPSS for Mac OS X (version 20.0; IBM® SPSS Inc., Chicago, IL, USA).

## Results

### Descriptive statistics

Out of the total 180 water samples from the 60 farms sampled in the study, a total of 20 (11.11%, 95% CI = 7.31–16.54) samples from nine farms were positive for *B. pseudomallei* while the remaining 160 (88.89%, 95% CI = 83.46–92.69) samples from 51 farms were negative (Table 1). The highest frequency of isolation was observed from boreholes and wells. However, there was no significant difference between the presence of the *B. pseudomallei* in water sample and source of water (underground or surface) (*χ*^*2*^ = 3.40, df = 1, OR = 2.52, 95% CI = 0.92–6.87, *p* = 0.065). There were also no significant associations between the presence of *B. pseudomallei* and farm management system (*χ*^*2*^ = 2.14, df = 2, *p* = 0.34) or the type small ruminants (goats, sheep, or mixed) kept in the farm (*χ*^*2*^ = 5.65, df = 2, *p* = 0.059).

**Table 1:** The sources of the water samples from the small ruminant farms in Peninsular Malaysia and *B. pseudomallei* isolation

	Borehole *n* (%)	Dam *n* (%)	Pond *n* (%)	Pool *n* (%)	River *n* (%)	Tap *n* (%)	Well *n* (%)	Total
Negative	44 (86.3)	6 (100)	6 (100)	6 (100)	24 (92.3)	41 (91.1)	33 (82.5)	160 (88.9)
Positive	7 (13.7)	0 (0.0)	0 (0.0)	0 (0.0)	2 (7.7)	4 (8.9)	7 (17.5)	20 (11.1)
Total	51 (100)	6 (100)	6 (100)	6 (100)	26 (100)	45 (100)	40 (100)	180 (100)

Table 2 shows the frequency of isolation of *B. pseudomallei* from the water samples from farms in the study according to states sampled. The highest frequency of isolation was observed in samples from Pahang. However, chi-squared test showed no significant association between isolation of *B. pseudomallei* and states sampled in this study (*χ*^2^ = 0.84, df = 3, *p* = 0.83).

**Table 2:** The frequency of isolation of *B. pseudomallei* from water samples from small ruminant farms (*N* = 60) in Peninsular Malaysia according to states

	N. Sembilan *n* (%)	Pahang *n* (%)	Perak *n* (%)	Selangor *n* (%)	Total *n* (%)
Negative	28 (84.8)	54 (90.0)	49 (90.7)	29 (87.9)	160 (88.9)
Positive	5 (15.2)	6 (10.0)	5 (9.3)	4 (12.1)	20 (11.1)
Total (%)	33 (18.3)	60 (33.3)	54 (30.0)	33 (18.3)	180 (100)

### Univariable analysis of water parameters

Univariable analyses of pH values, dissolved oxygen (DO_2_) contents, optical densities (OD), chemical oxygen demand (COD), and temperatures of *B. pseudomallei*-positive and negative water samples are shown in Table 3. The analysis of the water parameters using independent *t* test found significant differences (*p* < 0.05) between the means of pH value, COD, and optical density of the *B. pseudomallei*-positive and those of the negative water samples (Table 3). There were however no significant differences between means of the dissolved oxygen (DO_2_) contents and temperatures of the positive and negative water samples.

**Table 3:** The *t* test results comparing means of pH, dissolved oxygen (DO_2_), optical density (OD), temperature, and chemical oxygen demands (COD) of water samples from small ruminant farms in Malaysia

Parameter	Negative (*n* = 160) Mean ± SD	Positive (*n* = 20) Mean ± SD	*t* statistic	*p* value
pH	5.00 ± 0.58	6.21 ± 0.95	− 5.88	0.005*
DO_2_ (%)	10.98 ± 2.89	11.15 ± 3.82	0.24	0.81
OD (%)	0.25 ± 0.12	0.41 ± 0.05	− 10.14	< 0.001*
Temp (°C)	29.26 ± 4.00	29.80 ± 3.74	− 0.58	0.56
COD (mg/l)	25.48 ± 7.28	32.11 ± 5.05	− 8.25	< 0.001*

*There is a significant difference between the means at *p* < 0.05 level

*n* number sampled, *SD* standard deviation

### Multivariable logistic regression

Five variables (small ruminant type (goat, sheep, or mixed), source of water supply (underground or surface), and the water properties (pH, DO_2_ content, and COD)) were included in the logistic regression analysis. Table 4 shows the final model from the multivariable logistic regression analysis of the variables associated with the presence of *B. pseudomallei*. The Hosmer and Lemeshow test indicated that the model was a good fit for the data (*χ*^*2*^ = 7.13, df = 8, *p* = 0.52). Only the physicochemical properties of pH and COD remained significant as predictors for the presence of *B. pseudomallei* in water samples from the study farms when effects of other variables were taken into account. The odds of isolation of *B. pseudomallei* were significantly higher in water samples with higher pH value (OR = 8.12, 95% CI = 3.73–17.68, *p* < 0.001) and higher COD (OR = 1.002, 95% CI = 1.001–1.003, *p* = 0.004) compared with samples with lower pH and COD respectively.

**Table 4:** Water parameters significantly associated with occurrence of *B. pseudomallei* in water samples based on logistic regression analysis

Parameter	Descriptive *n* = 180 Mean ± SD (95%CI)	Negative *n* = 160 Mean ± SEM	Positive *n* = 20 Mean ± SEM	Odds ratio (OR)	OR 95% CI	*p* value
pH	5.13 ± 0.73 (5.04–5.25)	4.99 ± 0.57	6.21 ± 0.90	12.69	2.67–60.35	0.001*
COD	26.22 ± 7.35 (25.13–27.28)	25.48 ± 7.28	32.11 ± 5.05	1.002	1.001–1.003	0.004*

## Discussion

The relatively higher frequency of *B. pseudomallei* isolation observed in samples from the wells and boreholes in this study was probably because water from these sources has not been filtered or treated (Baker et al. 2011). Contaminated water has been incriminated as a source of infection to farm animals and humans in the melioidosis endemic areas (Ketterer et al. 1986; Inglis et al. 2000; Limmathurotsakul et al. 2014). In an event of flood, the contaminated water may facilitate dissemination of the agent to other areas (Chuah et al. 2017) spreading the infection. We found a significant difference between the means of pH value of *B. pseudomallei*-positive compared to that of the negative water samples where more acidic pH among the negative samples appear to be less preferred as compared to higher pH of the positive samples. Our observation is consistent to that of Chen et al. (2003) who reported the preferred pH for *B. pseudomallei* growth increase as pH increased from 5 until 7 with optimal growth range at pH 6.5 to 7.5. This was further supported by the Draper et al. (2010) study in northern Australia that also found slightly acidic water pH of 6.3 and 6.5 were preferred by the bacteria (Draper et al. 2010).

The COD is an indirect measure of organic matter contents in water samples and is considered to be a useful measure of water quality (Riedel et al. 1988). The COD is commonly used to determine the concentration of organic pollutants in water samples (Da Silva and Sacomani 2001) and is directly correlated with turbidity (Nguyen et al. 2014). This study found that positive water samples have a significantly higher mean of COD, consistent with those of Palasatien et al. (2008) in soil samples of northeast Thailand. In addition, Draper et al. (2010) found higher turbidity in water samples in northern Australia to significantly associate with the presence of the bacteria. There is very little information and explanation on the effect of COD on bacterial survival and growth; therefore, we find this finding difficult to explain. It is possible that the higher amount of organic compounds in the positive samples improves the survival of this saprophytic bacteria (Draper et al. 2010; Palasatien et al. 2008); however, the mechanics of this need further investigation.

We did not find significant differences between means of dissolved oxygen (DO_2_) contents of *B. pseudomallei*. The DO_2_ content indicates the amount of oxygen present in water sample and is usually influenced by temperature, quality of sediments in the water, and rate of utilization and replacement of the oxygen in the water (Sánchez et al. 2007). Other studies have shown that dissolved oxygen content supports growth of *B. pseudomallei* since depletion of oxygen content of water resulted in slowing of growth of the agent in liquid medium (Hamad et al. 2011). Similarly, our results did not show significant differences between means of the temperatures of *B. pseudomallei*-negative and *B. pseudomallei*-positive water samples. However, other study has reported that temperature of liquid media affects the growth of *B. pseudomallei* (Pitt 1995).

In conclusion, this study found that the pH and COD of water were the two most important physicochemical parameters associated with occurrence of *B. pseudomallei* in water sources from small ruminant farms from Peninsular Malaysia. The information on the relationship between these parameters and occurrence of the agent in water in Malaysia may serve as input in planning of control strategies against exposure to *B. pseudomallei* from this important environmental reservoir of the agent.
